# Avian Predation in a Declining Outbreak Population of the Spruce Budworm, *Choristoneura fumiferana* (Lepidoptera: Tortricidae)

**DOI:** 10.3390/insects12080720

**Published:** 2021-08-11

**Authors:** Jacques Régnière, Lisa Venier, Dan Welsh

**Affiliations:** 1 Natural Resources Canada, Canadian Forest Service, Laurentian Forestry Centre, 1055 rue du PEPS, Quebec City, QC G1V 4C7, Canada; 2 Natural Resources Canada, Canadian Forest Service, Great Lakes Forestry Centre, 1219 Queen St. E., Sault Ste. Marie, ON P6A 2E5, Canada; lisa.venier@canada.ca; 3 Environment and Climate Change Canada, Canadian Wildlife Service, Ottawa, ON K1A 0H3, Canada

**Keywords:** spruce budworm, predation, birds, warblers, exclosure cages, foraging

## Abstract

**Simple Summary:**

Cages preventing access to birds were used to measure the rate of predation by birds in a spruce budworm population during the decline of an outbreak. Three species of budworm-feeding warblers were involved in this predation on larvae and pupae. It was found that bird predation is a very important source of mortality in declining spruce budworm populations, and that bird foraging behavior changes as budworm prey become rare at the end of the outbreak.

**Abstract:**

The impact of avian predation on a declining population of the spruce budworm, *Choristoneura fumifereana* (Clem.), was measured using single-tree exclosure cages in a mature stand of balsam fir, *Abies balsamea* (L.), and white spruce, *Picea glauca* (Moench.) Voss. Bird population censuses and observations of foraging and nest-feeding activity were also made to determine the response of budworm-linked warblers to decreasing food availability. Seasonal patterns of foraging. as well as foraging success in the declining prey population was compared to similar information from birds observed in another stand where the spruce budworm population was rising. Avian predation was an important source of mortality between the 4th instar and moth emergence in the declining outbreak population. Mortality by predation increased from negligible to over 98% as budworm density dropped from 100 to <1 larva/kg of host foliage, over 3 years. Calculations based on nest-feeding activity and basic metabolic demands support these observed rates. Seasonal and yearly differences in predation rates observed between the two host-tree species correspond to equivalent shifts in bird foraging behavior in response to dropping insect density. In particular, a preference for searching on white spruce disappeared, although budworm-linked birds remained more efficient at finding food on this plant. The ability to change foraging behavior as prey density dropped differed between bird species.

## 1. Introduction

The spruce budworm (SBW), *Choristoneura fumiferana* (Clem.), is the most important herbivore disturbance in eastern North American boreal forests [1]. It is a periodic grazing disturbance (*sensu* [2]) that occurs over wide geographical areas at intervals of 30–40 years [3]. Birds are an important component of temperate forest ecosystems, acting as predatory regulators of many forest insect herbivores [4]. Following earlier authors [5,6], Morris [7] suggested that avian predation was responsible for keeping populations of the spruce budworm at an endemic density between outbreaks. He hypothesized that because birds are vertebrate predators, they probably exhibit a Type III (sigmoidal) functional response [8] that would produce a low-density depression in SBW population growth rates, the so-called “predator pit”. This depression would create a lower-equilibrium density around which SBW populations fluctuate until stand conditions, climate, or immigration trigger an outbreak. During outbreaks, SBW populations are limited by food supply. This type of population regulation has been labeled a multiple-equilibrium system [9,10].

Royama [11] rejected that hypothesis because he saw in existing field data no evidence of dichotomy in behavior between low and high spruce budworm population levels. Rather, he concluded that population cycles were the result of time-delayed (i.e., high-order), density-dependent larval mortality. By elimination, Royama suggested that a complex of diseases and parasites was responsible for the gradual change in larval mortality associated with the outbreak cycle [11]. This has been amply confirmed by more recent studies, and it has become evident that different natural enemies play different roles at different points in the outbreak cycle [12,13,14]. Predation (probably by birds) played an important role during the collapse of the 1980s spruce budworm outbreak in New Brunswick [13].

Birds are considered generalist predators obeying rules of optimal foraging [15]. As such, these predators should not be interested in hunting rare, unprofitable prey and therefore should not track low-density spruce budworm populations. However, there is ample evidence that populations of budworm-linked migratory birds respond to changes in spruce budworm abundance [16,17,18,19,20,21]. These birds, in particular the Tennessee warbler *Leiothlypis peregrine* (Wilson) [22], the Cape May warbler *Setophaga tigrina* (Gmelin) [23], and the bay-breasted warbler *S. castanea* (Wilson) [24] are conifer gleaners that use the spruce budworm as a major food source during the critical nesting period, in mid to late June.

In the mid-1980s, as a massive SBW outbreak in eastern North America was declining, we hypothesized that avian predation should play an important role in accelerating the decline of outbreak-level spruce budworm populations, once initiated by other factors. This would occur because of the increased numbers of birds with budworm-oriented feeding habits that responded to earlier increases in budworm. We measured the impact of bird predators on a declining spruce budworm population using exclosure cages and made observations on the density and foraging activity of budworm-linked warblers in the same mixed-wood forest stand. We compared those observations with data obtained in a second, distant stand with similar characteristics where budworm populations had just reached outbreak levels. We waited until now to present those data because we first needed to gain a better global understanding of the role of natural enemies in the dynamics of spruce budworm populations [12,13,14]. We also wanted to ensure that the SBW outbreak decline observed in the late 1980s in eastern North America was indeed the end of that outbreak rather than a temporary relapse.

## 2. Materials and Methods

### 2.1. Spruce Budworm Biology

The spruce budworm is a univoltine moth species. At latitudes and elevations covered in this study, adults fly in mid-July. Eggs are laid and hatch within a few weeks. By mid-August, hatchlings have spun silk shelters called hibernacula in bark crevices and other small structures in the tree crowns where they molt to a second instar and enter diapause for the winter. In early May, the overwintered larvae emerge from the hibernaculum and find host foliage to feed on the growing shoots. The feeding larvae undergo an additional four instars and pupate in late June. Moths emerge a few weeks later, and the cycle starts over again. As they grow from the 4th instar to the pupa, the weight and caloric content of budworm increases by a factor of 100 [25].

### 2.2. Study Plots 

**Figure 1:**
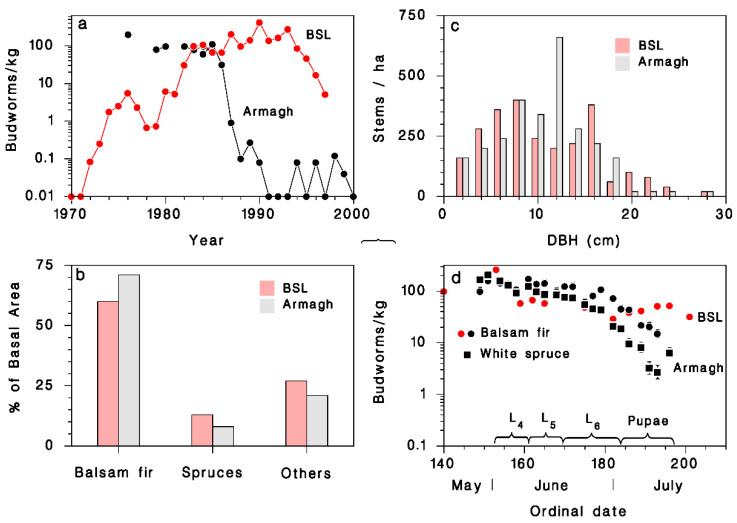
History of budworm outbreaks and description forest stands at Black Sturgeon Lake (BSL) and Armagh. (**a**) Population density at the 4th instar (BSL: red; Armagh: black). (**b**) Basal area of balsam fir, spruces (white and black), and other species. (**c**) Distribution of balsam fir diameters at breast height (DBH, cm). (**d**) Budworm population density during their summer development at BSL on balsam fir in 1983 (red) and on balsam fir and white spruce in Armagh in 1985 (black). Seasonal development of budworm is indicated along the date axis in (**d**). Data from [12,28].

Most of the work reported here was done in a conifer stand in the foothills of the Appalachian Mountains on the south shore of the St-Lawrence River near Armagh, Quebec, Canada (46.766° N, 70.657° W), between 1985 and 1988. Comparable information was also obtained in 1983 in a boreal mixed wood stand near Black Sturgeon Lake (BSL; 49.329° N, 88.893° W) north of Lake Superior in northwestern Ontario, more than 1400 km to the northwest of Armagh. These plots were chosen because of their contrasting spruce budworm population history at the time of the study [12,26]. The budworm population at BSL reached damaging levels in 1982 after a gradual climb from low densities in the early 1970s (red, Figure 1a). In 1983, when the data reported here were collected, the budworm population at BSL was at 96 larvae/kg of host foliage, a density expression that facilitates comparison between host species and locations [27]. In Armagh, budworm populations had been at outbreak levels since 1975 and started to decline in 1985, when density at the 4th instar was 110 larvae/kg (black, Figure 1a). By 1988, population density had dropped four orders of magnitude to 0.1 larva/kg. The two stands had similar makeups in terms of dominant tree canopy [28] (Figure 1b). Balsam fir (*Abies balsamea* L.) was the most abundant tree species (60–70% of basal area). There was a significant amount of white spruce, *Picea glauca* (Moench) Voss. in both stands, with black spruce *P. mariana* (Mill.) Britton also present at BSL (spruces represented 8–13% of stems). Aspen, *Populus tremuloides* Michx., was the dominant deciduous tree in both plots, but was accompanied in Armagh by red maple, *Acer rubrum* L., and white birch, *Betula papyrifera* Marshall (21–27% of stems). Balsam fir trees in Armagh and at BSL had the same 10.7 cm average diameter at breast height (DBH) (Figure 1c). The climate in Armagh is slightly warmer and wetter (3.8 °C mean annual temperature; 2575 annual degree-days > 0 °C, 1016 annual precipitation) than at BSL (1.7 °C mean annual temperature, 2320 annual degree-days > 0 °C, 793 mm annual precipitation); adult emergence of budworm occurs one week later at BSL than in Armagh [28]. The seasonal change in population density observed over the summer development period illustrates the contrasting level of mortality in rising populations (at BSL in 1983) and declining populations (in Armagh in 1985) (Figure 1d).

### 2.3. Observations on the Spruce Budworm

#### 2.3.1. Exclosure Cages

**Figure 2:**
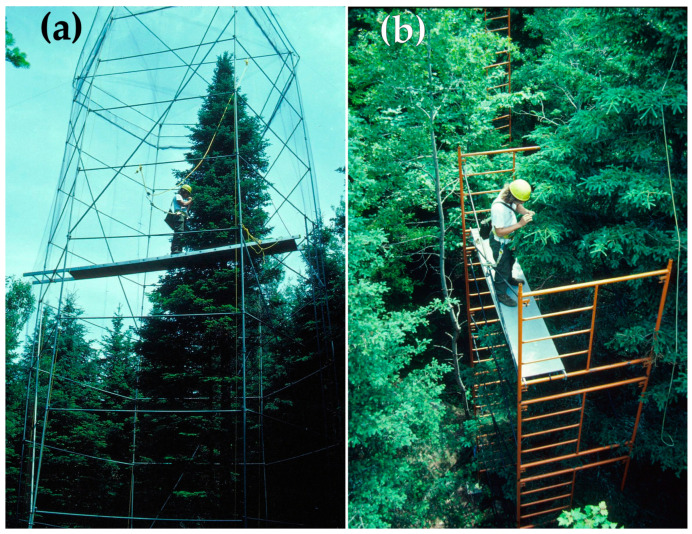
Implanted budworm cohort observations being recorded at mid-crown on mature white spruce trees in Armagh in 1987. (**a**) Inside a bird exclosure cage. (**b**) On scaffolding.

The impact of avian predation on spruce budworm populations between the 4^th^ instar and moth emergence was estimated in Armagh only, from 1986 to 1988, by measuring the difference in mortality inside and outside of exclosure cages (Figure 2). The cages, built around 8 single, mature trees (4 balsam fir and 4 white spruce) were 15–20 m-high, 6.7 m-diameter hexagonal frames of 2–2.5 cm steel pipe connected with aluminum clamps (Gascoigne Industrial Products, Buffalo, NY, USA). A fine black-nylon net, 1.5 cm-mesh (Quest Plastics Co., Mississauga, ON, Canada) denied birds access to the trees inside each cage. Nets were installed in early May, after the dispersal period of 2nd instar spruce budworm larvae emerging from diapause, and were removed just prior to the period of egg hatch in late July. The nylon mesh of the nets was very thin and could not have created a microclimate around caged trees. Because the net was not buried at the base of cages, its presence probably did not prevent the passage of other potential vertebrate predators. 

#### 2.3.2. Population Sampling 1986 

In 1986 in Armagh, when budworm populations were still high, 45-cm branch tips were taken at random every 2–4 days between the 3rd instar and adult emergence from the mid crown of dominant and co-dominant balsam fir and white spruce trees (12–25 of each species) with pole pruners equipped with collecting baskets [29]. On each sample date, until the sixth instar, two branch tips were also collected from the mid-crown of each of the eight caged trees. The number and development stage of spruce budworm in these samples were determined in the laboratory, and insect density was expressed per kg of foliage [12]. However, after the beginning of the 6th instar, branch tips were no longer severed from caged trees to conserve foliage, and the branch tips were examined in situ. In this case, the average weight of samples taken earlier inside the cages was used to calculate density.

#### 2.3.3. Implanted Cohorts 1987

After 1986 in Armagh, budworm populations dropped to densities that were too low to be monitored by random sampling of host foliage, because sample sizes needed would have been prohibitively large (see Figure 1a). Instead, we used implanted insects. In 1987, spruce budworm larvae from a laboratory colony (Stock: Glfc:IPQL:Cfum; Sault Ste. Marie, ON, Canada; [30]) were installed, using a fine paintbrush, on growing shoots at mid-crown on the 8 caged trees as well as on 10 nearby, uncaged trees (5 balsam fir, 5 white spruce). Four cohorts (on balsam fir and white spruce, inside and outside of the cages) were implanted in the 3rd instar (*n* = 1070), and four more in the early 6th instar (*n* = 653). Timing of the installation of these cohorts was determined with a phenology model [31], calibrated with data from wild insects collected at emergence and around peak 4th instar. A transparent-mica label (0.5 × 2 cm) bearing an identification number, was pinned on a twig within 10-cm of each larva, using inconspicuous black, brown, or green map pins. Larvae were checked every 2–4 days until dead or lost, and their development stage was noted, whenever possible without disturbing them. 

#### 2.3.4. Implanted Cohorts 1988

In 1988, we modified the implantation technique to use feral insects and avoid the installation of two successive cohorts. Budworm pupae were collected at the end of the 1987 season in high-density spruce budworm populations in Québec. Egg masses were placed in plastic vials, with balsam fir flower-scar clusters stripped of their needles [32]. Two weeks after the hatched larvae had spun hibernacula (early August 1987), these flower clusters were installed on the underside of mid-crown branches inside and outside the exclosure cages, on the same trees used in the 1987 season. These implanted insects overwintered and established naturally on growing shoot in early May 1988. The foliage was then searched in situ, and larvae found within the first two weeks constituted the four cohorts (*n* = 1839). The established larvae were inspected at 2–4 day intervals. On each visit, the number of live spruce budworms found on the foliage was recorded.

#### 2.3.5. Data Analysis 

The measurements of SBW density made in 1986 provided four time series consisting of successive independent random foliage samples. Assuming that the only difference between caged and uncaged individuals is the influence of avian predation, we can write: *N_u_*(*t*) = *N_c_*(*t*) × *S_p_*(*t*), where *N_u_*(*t*) and *N_c_*(*t*) are the density of uncaged and caged SBW at time *t*, respectively, and *S_p_*(*t*) is the survival from predation up to that time. Thus, an estimate of survival from predation is

*S_p_*(*t*) = *N_u_*(*t*)/*N_c_*(*t*) (1)

These sequential estimates of predation survival were submitted to analysis of variance-covariance with a general linear model (GLM) analysis using the model:

log_10_[*S_p_*(*t*)] = *p*_0*i*_ + *p*_1*i*_*t* + *p*_2*i*_*t* + ε (2)

where *i* is the host tree species index (either balsam fir or white spruce) and *t* is time, in days since the first sample, and ε is a residual (error) term. The model was simplified by eliminating the least significant terms one at a time until all remaining terms were either significant (α = 0.05) or needed for hierarchical integrity. Residuals were tested for normality using the Anderson-Darling test [33].

In 1987 and 1988, cohorts of implanted insects were used instead of random foliage samples. Because of this, the numbers of insects alive at each successive date *t* are not independent (a drop in numbers on a given date leads to reduced numbers on all subsequent dates). To circumvent this sequential dependence, the rate of survival between successive sample dates was calculated as the ratio *s*(*t*) = *N*(*t*)/*N*(*t* − 1) where *N*(*t*) is the number of SBW alive at time *t* and *N*(*t* − 1) the number alive on the previous date. We submitted these survival rates to binary logistic regression using the model:

(3) $$\ln\left[\frac{s_{ijk}(t)}{1-s_{ijk}(t)}\right]=p_{0ijk}+p_{1ijk}t$$

where *i* is the host tree species index (balsam fir or white spruce), *j* is the cage index (in or out), *k* is the cohort index (L_3_ or L_6_, used in 1987 only), and *t* is time, in days since the first sample. Twice in the early season of 1988, more individuals in a cohort were recorded than on the previous inspection date. Those two observations were omitted from the analysis. Equation (3) was reduced by removing the least significant terms one at a time. Reduction stopped as soon as the AICc was minimized.

The partial product of sequential estimates of survival rates predicted with Equation (3) was then used to calculate expected cumulative survival over time *S*(*t*), on balsam fir and white spruce (index *i*), in and out of exclosure cages (index *j*):

(4) $$S_{ij}(t)=\prod_{t}s_{ij}(t)$$

Survival from predation *P*(*t*), either observed or predicted, was then calculated as the ratio of survival outside *S_i_*_0_(*t*) to inside cages *S_i_*_1_(*t*):

(5) $$P_{i}(t)=\frac{S_{i0}(t)}{S_{i1}(t)}$$

where the observed survival is the ratio of number alive at time *t* to initial number at the beginning of the time series: *S_ij_* (*t*) = *N_ij_* (*t*)/*N_ij_* (0).

### 2.4. Observations on Birds 

Although data were gathered on a larger number of bird species, the analysis here is concentrated on three warbler species commonly regarded as spruce budworm-linked: bay-breasted (BBWA), Cape May (CMWA), and Tennessee (TEWA). We also include data on the Blackburnian warbler (BLBW), *Setophaga fusca* (Müller), a species with some previous evidence of response to budworm [19], although this may be more the result of population movements [34] and territory-size adjustments [35] than actual numerical response. 

#### 2.4.1. Population Censuses 

Songbird populations were censused in late-May, early-June in Armagh (1986–1988) and BSL (1983) by standard auditory, unlimited-distance, 10 min point counts [36] in the plots (roughly a 100m radius). Censuses were conducted on several days during the nesting period each year. In Armagh, a second series of censuses was conducted in the forested area surrounding the budworm sampling plot. The number of pairs of each species recorded in the censuses was transformed to rough estimates of density per km^2^ assuming that the census points effectively sampled a circle of 100 m radius.

#### 2.4.2. Foraging Activity

Foliage-gleaning birds were observed with binoculars from scaffolds erected in small clearings at BSL in 1983, and in Armagh in 1987. Observations were made throughout daylight hours from late May to early July. In Armagh (1987), 5951 observations were recorded in 403 series (sighting and following a given individual) over 251 h of observation. At BSL (1983), a total of 7552 records were made in 218 series over 140 h. The tree species on which the birds were seen, and their activity (vocalization, flight, searching, probing, etc.) was recorded. Probing with the beak was considered a successful search or feeding.

#### 2.4.3. Nesting Activity 

In Armagh in 1987, the average time between visits to the nest by a pair of bay-breasted warblers raising 5 young was recorded, on 7 of the 13 days during which the adults fed the young. The daily frequency of visits to the nest by either parent was calculated assuming a 16 h activity period because there was no obvious pattern of visits during the daylight hours. Each time a bird was observed returning to the nest, an attempt was made to estimate the number of food items it was carrying. Most of the time, this was impossible. On average, the parent returned to the nest with 5.5 prey items, which included larvae, pupae, or moths of spruce budworm and other insects.

#### 2.4.4. Data Analysis 

Bird population density data were analyzed within species, by ANOVA after square-root transformation, with an Anderson-darling test of normality of residuals [33]. Counts of foraging observations were compared by chi-square tests. Bird foraging success on balsam fir, spruces, and deciduous trees was contrasted by Cochran-Mantel-Haenszel tests [37] after Bonferroni multiple-comparison adjustment.

## 3. Results

### 3.1. Observations on Spruce Budworm 

**Figure 3:**
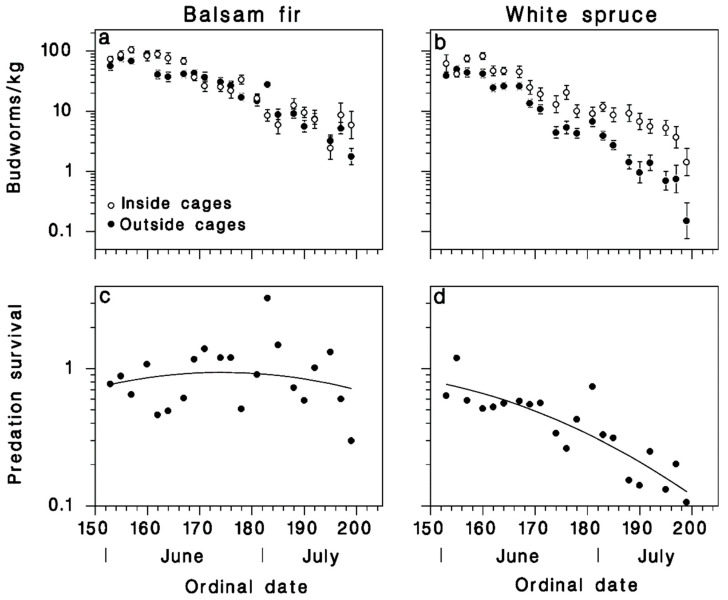
Seasonal changes of spruce budworm density and survival from predation in Armagh in 1986 on (**a**,**c**) balsam fir and (**b**,**d**) white spruce (means ± SEM). (**c**,**d**) symbols: observed survival from predation (Equation (1)), lines: survival predicted with Equation (6).

The rising spruce budworm population of BSL in 1983 had a budworm density of 96 larvae/kg, almost identical to the 110 larvae/kg in Armagh at the onset of outbreak decline in 1985 (Figure 1a). At BSL in 1983, mortality of large larvae and pupae was low (Figure 1d). Mortality among late larval stages and pupae was much higher in the declining outbreak population of Armagh in 1985, where it was also considerably higher on white spruce than on balsam fir (Figure 1d). In 1986 at Armagh, based on a comparison of SBW numbers inside and outside cages, there was a significant amount of avian predation, on white spruce (Figure 3b). 

The reduced model (Table 1)

(6) $$\log_{10}\left[S_{p}(t)\right]=\left\{ \begin{array}{ll} 0.1149+0.00818\;t-0.000192\;t^{2} & \mathrm{on}\;\mathrm{balsam}\;\mathrm{fir}\\ 0.1149-\;0.00818\;t-0.000192\;t^{2} & \mathrm{on}\;\mathrm{white}\;\mathrm{spruce} \end{array}\right.$$

indicates rapidly decreasing survival of SBW populations over time on white spruce, but no such trend on balsam fir. Mortality outside the cages in 1986 was very similar to that observed in 1985 on both host tree species (compare Figure 1d with Figure 3a,b). There was no evidence of avian predation on balsam fir in 1986, (Figure 3c), but end-of-season predation mortality reached 80% as calculated with Equation (6) on white spruce (Figure 3d).

**Figure 4:**
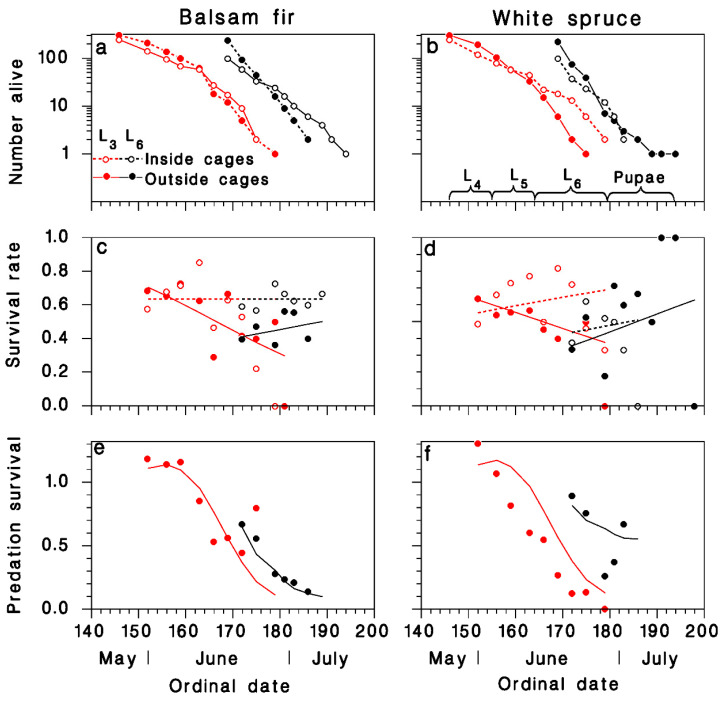
Survival among implanted cohorts in Armagh in 1987 on (**a**,**c**,**e**) balsam fir and (**b**,**d**,**f**) white spruce (red: L_3_ cohort; black: L_6_ cohort; Open circle: inside cages; closed symbols: outside cages). (**a**,**b**) number of live individuals. (**c**,**d**) Survival rate (lines: Equation (7); dotted: inside cages; solid: outside cages). (**e**,**f**) survival from predation calculated with Equation (5).

In 1987 at Armagh, mortality among the implanted cohorts inside and outside of the cages was higher (Figure 4) than among the naturally-occurring SBW in 1985 (Figure 1d) or 1986 (Figure 3a,b). While this may have resulted in part from handling losses because larvae were installed on foliage with brushes, especially in the L_6_ cohort (black lines and symbols in Figure 4), it probably also resulted from high levels of mortality inflicted by natural enemies. Survival among caged larvae was significantly higher than for uncaged larvae in the L_3_ cohorts, on both host species (red symbols and lines, Figure 4c,d; Table 2). This difference remained in the L_6_ cohort on balsam fir (black symbols and lines in Figure 4c), but not on white spruce (black symbols and lines in Figure 4d). These complex results are well described by regression Equation (3) after reduction (Table 2):

(7) $$\ln\left[\frac{s(t)}{1-s(t)}\right]=\left\{ \begin{array}{ll} 0.5524 & L_{3}\;\mathrm{on}\;\mathrm{balsam}\;\mathrm{fir}\;\mathrm{in}\;\mathrm{cages}\\ 1.224-0.05969\;t & L_{3}\;\mathrm{on}\;\mathrm{balsam}\;\mathrm{fir}\;\mathrm{outside}\;\mathrm{cages}\\ 0.08308+0.02142\;t & L_{3}\;\mathrm{on}\;\mathrm{white}\;\mathrm{spruce}\;\mathrm{in}\;\mathrm{cages}\\ 0.7544-0.03826\;t & L_{3}\;\mathrm{on}\;\mathrm{white}\;\mathrm{spruce}\;\mathrm{outside}\;\mathrm{cages}\\ 0.5524 & L_{6}\;\mathrm{on}\;\mathrm{balsam}\;\mathrm{fir}\;\mathrm{in}\;\mathrm{cages}\\ -0.9289+0.02166\;t & L_{6}\;\mathrm{on}\;\mathrm{balsam}\;\mathrm{fir}\;\mathrm{outside}\;\mathrm{cages}\\ -0.8173+0.02142\;t & L_{6}\;\mathrm{on}\;\mathrm{white}\;\mathrm{spruce}\;\mathrm{in}\;\mathrm{cages}\\ -1.714+0.04308\;t & L_{6}\;\mathrm{on}\;\mathrm{white}\;\mathrm{spruce}\;\mathrm{outside}\;\mathrm{cages} \end{array}\right.$$

The resulting estimates of survival from predation, produced by Equation (5), show that birds consumed a large proportion of the insects in both cohorts on balsam fir (Figure 4e). On white spruce, birds consumed nearly all larvae of the L_3_ cohort, but only about 50% of those in the L_6_ cohort (Figure 4f).

**Figure 5:**
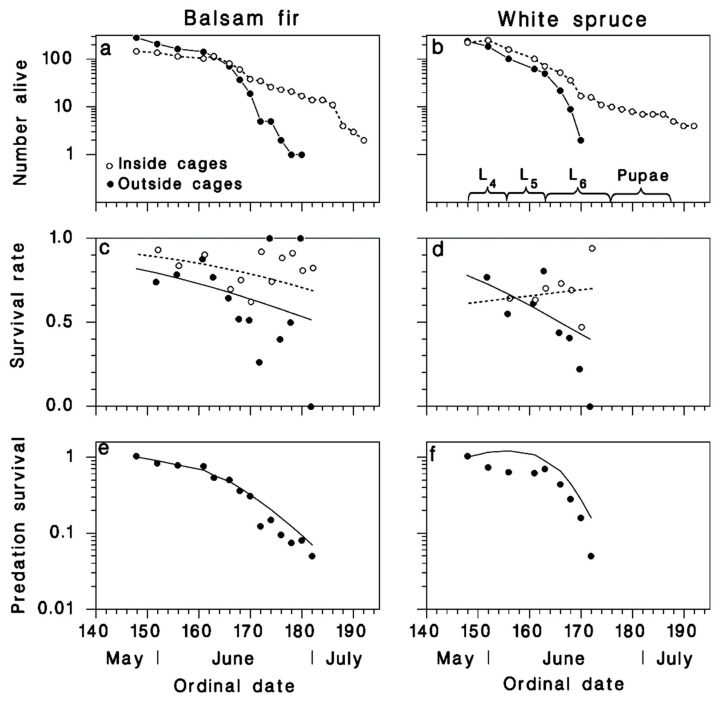
Survival among implanted cohorts in Armagh in 1988 on (**a**,**c**,**e**) balsam fir and (**b**,**d**,**f**) white spruce (Open circle: inside cages; closed cirles: outside cages). (**a**,**b**) number of live individuals. (**c**,**d**) Survival rate (lines: Equation (8); dotted: inside cages; solid: outside cages). (**e**,**f**) survival from predation calculated with Equation (5).

Results from the cohorts of 1988, implanted while in overwintering on caged and uncaged balsam fir and white spruce trees, were very similar to those of 1987 (Figure 5a,b). Survival rates were significantly lower outside than inside the exclosure cages, on both host species (Figure 5c,d; Table 3), and decreased as time progressed. The highest predation rates (>90%) occurred as larvae reached the late stages at the end of June. The reduced model yielded:

(8) $$\ln\left[\frac{s(t)}{1-s(t)}\right]=\left\{ \begin{array}{ll} 2.242-0.04292\;t & \mathrm{on}\;\mathrm{balsam}\;\mathrm{fir}\;\mathrm{in}\;\mathrm{cages}\\ 1.515-0.04292\;t & \mathrm{on}\;\mathrm{balsam}\;\mathrm{fir}\;\mathrm{outside}\;\mathrm{cages}\\ 0.4546+0.01638\;t & \mathrm{on}\;\mathrm{white}\;\mathrm{spruce}\;\mathrm{in}\;\mathrm{cages}\\ 1.262-0.06999\;t & \mathrm{on}\;\mathrm{white}\;\mathrm{spruce}\;\mathrm{outside}\;\mathrm{cages} \end{array}\right.$$

### 3.2. Observations on Birds

#### 3.2.1. Population Censuses

**Figure 6:**
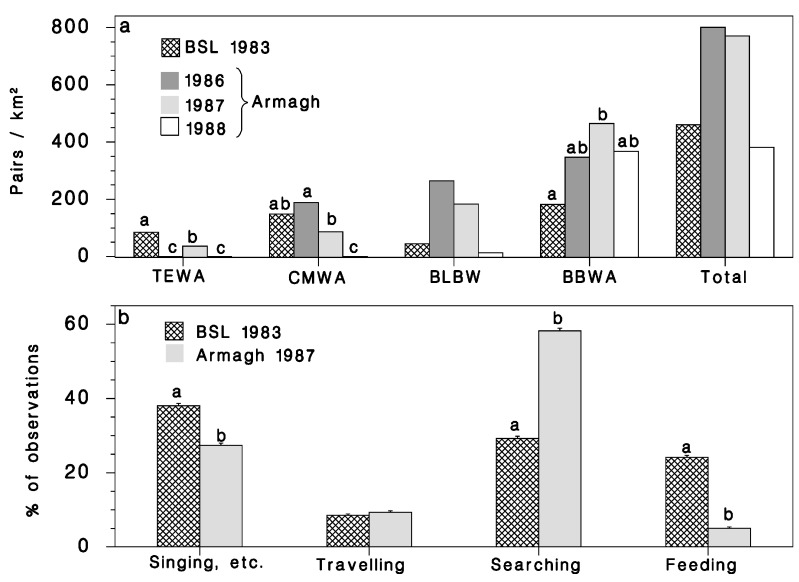
(**a**) Population density of four warbler species at Black Sturgeon Lake in 1983 (cross-hatched bars) and in Armagh in 1986, 1987, and 1988 (bars in decreasing shades of grey). Letters indicate Bonferroni mean-separation test results (within species). (**b**) Relative frequency of bird activity observations classified into four categories, at BSL in 1983 and Armagh in 1987. Letters indicate significant site differences (χ^2^ test of association) for each activity. Vertical lines: binomial standard error $\left(\sqrt{p(1-p)/n}\right)$.

Our data did not indicate significant differences in the total density of the three species of budworm-linked warblers and Blackburnian warbler at BSL in 1983 and in Armagh from 1986 to 1988 (Table 4; Figure 6a). However, there was significant variation in density within individual species. Tennessee warblers were more abundant at BSL than in Armagh and were not detected in Armagh in either 1986 or 1988. Cape May warblers were equally numerous at BSL and Armagh, and there was a significant decrease in the numbers from 1986 to 1988 in Armagh (Figure 6a; Table 4). The variability of Blackburnian warbler counts was too high to detect significant patterns in this species’ density. The density of bay-breasted warblers was highest, and they were significantly more numerous in Armagh (especially in 1987) than at BSL in 1983 (Figure 6a, Table 4).

#### 3.2.2. Foraging Activity 

The proportion of observations of budworm-linked warblers devoted to four activities (traveling, searching, feeding, singing, and others) was dramatically different between BSL in 1983 with 96 budworms/kg, and Armagh in 1987 with 1 budworm/kg (Figure 6b; Table 5). The birds at BSL spent much of the time vocalizing and were seen capturing prey almost as often as they were seen unsuccessfully searching. By contrast, birds in Armagh spent a large proportion of time searching for prey, with much less success. They also spent less time singing. 

**Figure 7:**
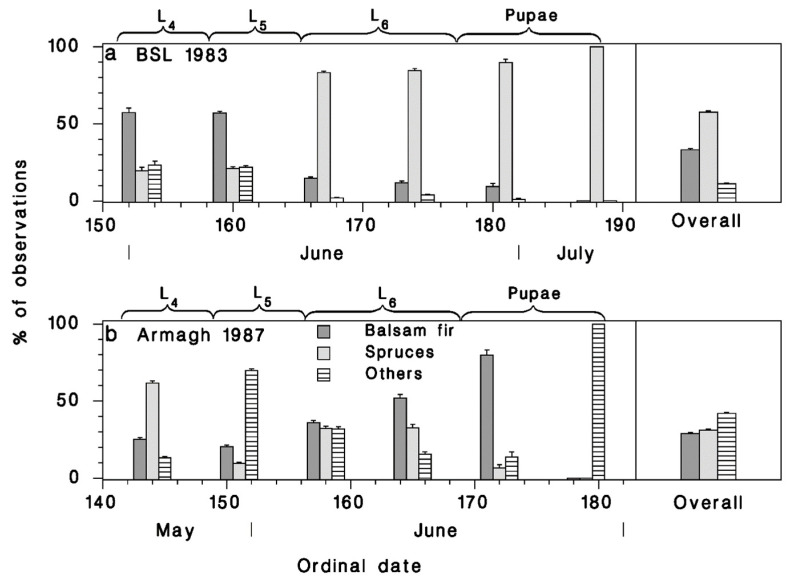
Relative frequency of observations recorded on various tree species, at (**a**) BSL in 1983 and (**b**) Armagh in 1987 over time in the summer and overall. Vertical lines: standard error $\left(\sqrt{p(1-p)/n}\right)$. Seasonal development of budworm illustrated above each temporal frame.

At BSL in 1983, budworm-linked species spent most of the nesting period (second half of June) foraging on spruce trees (Figure 7a). By contrast, the nesting birds in Armagh in 1987 foraged more on balsam fir and deciduous trees (Figure 7b). Overall, most foraging observations were made on spruces at BSL, and on deciduous trees in Armagh. 

**Figure 8:**
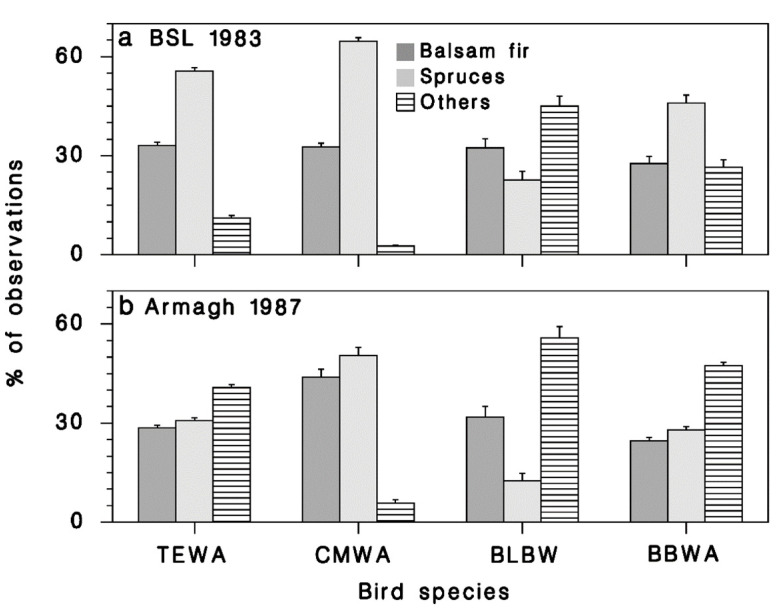
Relative frequency of observations recorded on various tree species, at (**a**) BSL in 1983 and (**b**) Armagh in 1987, gouped by bird species. Vertical lines: standard error $\left(\sqrt{p(1-p)/n}\right)$.

This difference in behavior was most pronounced in TEWA and BBWA (Figure 8). BLBW seemed to favor deciduous vegetation in both cases, while CMWA showed a strong preference for conifers in both plots.

**Figure 9:**
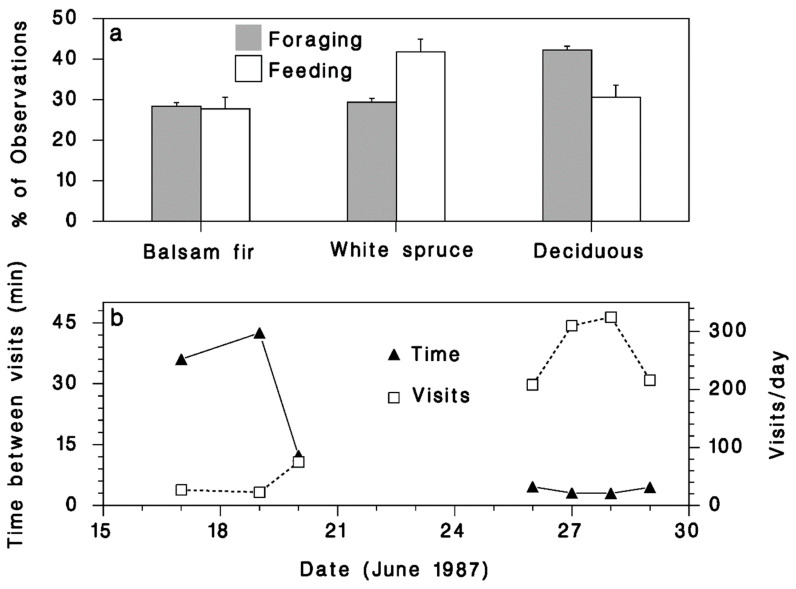
(**a**) Relative frequency of foraging (grey bars) and feeding (open bars) observations recorded on various tree species, in Armagh in 1987. Vertical lines: binomial standard error $\left(\sqrt{p(1-p)/n}\right)$. (**b**) Time between visits (closed triangles), and the number of visits per day (open squares), by a pair of BBWA raising five young in Armagh in 1987.

Foraging observations made in Armagh in 1987 suggest that the prey-finding efficiency of budworm-linked species varies significantly between host tree species (Figure 9a; χ^2^ = 22.5, df = 2, *p* < 0.001). Paired comparisons [37] indicate that the probability of probing (finding prey) is higher on white spruce than on either balsam fir (odds ratio = 1.5, CMH = 6.1, *p* = 0.014) or deciduous trees (odds ratio = 2.1, CMH = 21.5, *p* < 0.001), both significant differences using a Bonferroni-adjusted *α* = 0.025. Therefore, while budworm-linked birds spent most of their time searching for prey in deciduous vegetation (grey bars in Figure 9a) most of the successful feeding was observed on white spruce (open bars, Figure 9a).

#### 3.2.3. Nesting Activity

The average time between visits to the nest by the pair of BBWA adults decreased dramatically as the growing young birds demanded more food (Figure 9b). By the end of June, the adults made 200–320 daily visits to the nest, at intervals lasting less than 5 min. During the 13-day nesting period, the adults made an estimated 1804 foraging trips to feed their young, carrying an average of 5.5 food items on each trip. On this basis, raising the young probably required >9900 prey items.

## 4. Discussion

Mortality of the feeding larval and pupal stages of SBW was characteristically low in the rising outbreak population at BSL in 1983, and much higher at the onset of outbreak decline in Armagh in 1985 (Figure 1). These results highlight the fact that budworm outbreak collapse is associated with increased mortality in late larval instars (4th–6th) and pupal stage, linked to higher impact of natural enemies [11,12,13]. Our data suggest that the quantitative importance of avian predation increased dramatically as the spruce budworm population decreased between 1986 and 1988 in Armagh (black symbols in Figure 1a). However, these results must be interpreted prudently because our methods changed between years (from feral insect sampling in 1986 to installation of cohorts in 1987 and 1988), and because the use of implanted insects may have increased resource density at a very small spatial scale, especially in 1988. In 1986 (the first year of the cage study), avian predation mortality was significant on white spruce (Figure 3d) but was not detected on balsam fir (Figure 3c). Budworm mortality on white spruce inside the exclosure cages was the same as on balsam fir, caged or not (Figure 3a,b). Thus, avian predation was responsible for the mortality difference between the two host species. It is interesting to note that a similar difference in budworm survival between the two host species was observed in 1985 (black symbols in Figure 1d), which we believe may have been due to the same difference in avian predation pressure. However, as the budworm population declined from year to year (Figure 1d), this difference in predation mortality on budworm between host species disappeared (Figure 4 and Figure 5). Survival trends over summer suggest that most predation by birds occurred while larvae were in the late instars and pupal stage, in the second half of June (Figure 4e,f and Figure 5e,f).

Our data indicate that the availability of spruce budworm larvae in a low-density period is smaller than the food demand of the budworm-linked bird population. The caloric needs for maintenance and growth of a young warbler nestling between hatch and adult weight (11 g in these warblers) averages 14.9 Kcal/day [38]. Thus, a nest with 5 nestlings requires 970 Kcal of food over a 13-day period. The average caloric content of spruce budworm late-instar larvae and pupae is 0.104 cal/individual [25]. These energetic needs of the nestlings, therefore, represent a total of 9295 spruce budworm prey items pest nest. This compares very favorably with the estimated number of prey items brought to the nest (9922) calculated from the frequency of nest visits during the nestling growth period (1804) and the average number of prey items carried by parents at each visit (5.5). In Armagh in 1986 and 1987, the four warbler species had a total density of 8 pairs/ha (Figure 6b), which represents a total consumption of 74,364 budworm larvae and pupae just to raise their young. That number does not include the demands of adults, of transient birds, or of other bird species that consume spruce budworm, which is expected to be significant [19,39]. Crawford and Jennings [17] estimated from stomach content analyses that the same four species of warblers (but with the Nashville warbler instead of the Tennessee warbler) ate 78,610 budworms/ha. Régnière and Sanders (unpublished data) estimated that balsam fir and white spruce trees growing in a conifer forest stand had 3.1 kg of foliage/cm-DBH. Based on an average DBH of 11 cm in Armagh and a density of 2500 stems/ha, we estimate the amount of foliage around 82,250 kg/ha. At a late-stage density of 100 SBW/kg as at Black Sturgeon Lake in 1983, bird consumption represents only 1% of the SBW population. At 10 budworms/kg, as in Armagh in 1985 (Figure 2d), this proportion increases to 9%, and to 90% at a density of 1 budworm/kg as in Armagh in 1987 (Figure 1a). 

Our observations of bird abundance and foraging activity on the various tree species as their insect food source diminished help to understand these patterns. Part of the differences in the makeup of the budworm-linked bird community between Armagh and Black Sturgeon Lake may reflect habitat structure, which is more heterogeneous in Armagh [28]. Nevertheless, our data are consistent with the ample evidence that these birds are more numerous after several years of plentiful spruce budworm [39,40]. We believe the primary driver of predation response in this study is the difference in relative densities of spruce budworm and insectivorous bird populations.

Our results suggest that spruce budworm-linked warblers prefer to forage on white spruce when there is an overabundance of prey as evidenced by their preference for white spruce in BSL. This does not seem to be true for Blackburnian warblers, which were seen more often feeding on deciduous vegetation, regardless of spruce budworm density. This is consistent with the conclusions from [19] that TEWA, CMWA, and BBWA respond more strongly and consistently to budworm abundance than any other species. The reasons for higher foraging success on white spruce are not clear. White spruce branches are generally more rigid than balsam fir. Birds landing and moving on them in search of spruce budworm may be less readily detected by the larvae, which have a very distinct escape behavior, spinning down from their feeding site on a silk lifeline like many other lepidopteran larvae [41]. It is also possible that birds are better able to detect damaged white spruce shoots which are often curved and retain the bud cap for a longer time than unaffected shoots. Our evidence (Figure 9a) indeed suggests higher foraging success on white spruce. It is also interesting that the caloric value of sixth-instar budworm larvae and pupae is 10 to 15% higher on white spruce [25], which could also help explain the feeding preference from an optimal foraging standpoint.

It would seem that the rarity of spruce budworm larvae after outbreak decline has a large impact on the time budgets of predatory birds. Decreased prey availability implies higher foraging activity and a lower success rate. In response, some species may be sufficiently flexible to change foraging behavior and search for other prey, on other substrates. This seems to be the case for bay-breasted warblers that were observed foraging more on deciduous trees than spruce or fir. Others, like the Cape-May warbler, may not have this capacity: this species continued to forage mainly on spruce and fir in Armagh in 1987 when budworm density was low (Figure 8). This is consistent with findings from [40] that suggested the increase in bay-breasted warbler abundance persisted longer than the Cape-May Warbler. The consequences of a food supply drop for the latter may have been nest failure and starvation in mid-season in 1987. Increased competition for food on deciduous vegetation resulting from a shift in the foraging substrate of bay-breasted warblers may be the reason for a decrease in Blackburnian warbler numbers in 1988. Another factor that may contribute to the differential response of warblers over time is the impact of defoliation on foraging and nesting success [40]. Cape-May and Tennessee warblers tend to forage on the tops of trees and outer branches [22,23] where budworm defoliation is expected to be most severe, whereas bay-breasted warbler forages closer to the trunk [24].

## 5. Conclusions

The issue at this point is how long avian predation remains an important mortality factor in a declining spruce budworm population. Indeed, this may well determine how low populations of the insect are driven before predation pressure eases off. That undoubtedly depends on several factors, but perhaps most importantly on the mix of species in the predator community, on the numbers and diversity of non-budworm prey, as well as on the structure of the surrounding habitat [42]. The size and distribution of habitat patches have considerable impacts on the dynamics of ecosystems in response to disturbances [43,44]. Compared to boreal habitats, meridional forests generally have a more heterogeneous structure, with a more diverse mix of dominant plant species resulting in part from human activity. Consequently, the phytophagous insect fauna is also more diversified in these areas. Meridional bird communities should also tend to be dominated by birds that have the behavioral plasticity required to exploit a more varied food supply. The response of the bird community to a decrease in spruce budworm availability in more diverse meridional forests should therefore be prey switching, with consequent easing-off of predation pressure early in the decline. In simpler budworm-prone boreal forest stands, balsam fir and spruce budworm dominate the vegetation and herbivorous insect fauna. There, prey-switching may not be a feasible response, and the alternatives are movement or starvation after exhaustion of the food supply. This implies intensified predation pressure on spruce budworm. As a result of these differences, it is likely that the densities reached during the “endemic” period of the spruce budworm cycle are higher in meridional than in boreal forests. This implies that other natural enemies, particularly budworm-specific parasitoids, may remain more common in the more diverse habitats [45,46,47]. This lends support to the “enemies hypothesis” [48]. Thus, the complex of natural enemies should be in a position to react sooner to a recovery of spruce budworm population in habitats where bird predators have been able to switch prey earlier in the decline. Some, but not all, budworm-linked bird species seem to respond very early to a rising population of their budworm prey [19,21,40]. Recent observations of the density dependence of SBW survival in the larval and pupal stages in rising outbreak populations [14] support Morris’ [7] “predator pit” hypothesis. Prey-switching would lead to less pronounced outbreak cycles, with a somewhat higher intrinsic frequency in more diverse meridional forests, a conclusion that is in accordance with the findings of [49], as discussed by [50].

## Figures and Tables

**Table 1:** Analysis of variance for the effects of time (*t*, ordinal date) as a covariate, and host species (*s*) on spruce budworm population survival from predation in Armagh 1986 (Equations. (1) and (2)) (R^2^ = 0.66). Regression residuals were normally-distributed (AD = 0.37, df = 42, *p* = 0.42). The reduced model is Equation (6).

	Full Model			Reduced Model		
Source	df	F	*p*	df	F	*p*
*s*	1	1.48	0.519			
*t*	1	1.47	0.234			
*s* × *t*	1	3.44	0.072	1	41.05	<0.001
*t* ^2^	1	5.50	0.025	1	5.56	<0.001
*s* × *t*^2^	1	0.64	0.428			
Error	36			39		

**Table 2:** Results of binary logistic regression of survival (Equation (3)) among cohorts in Armagh in 1987 (*s*: host species; *ca*: presence of exclosure cages; *co*: cohort, either L_3_ or L_6_; *t* ordinal date). The reduced model is Equation (7).

	Full Model			Reduced Model		
Source	df	χ^2^	*p*	df	χ^2^	*p*
Regression	15	158.67	0.000	8	156.15	<0.001
*s*	1	4.73	0.030	1	11.86	0.001
*ca*	1	6.00	0.014	1	21.95	<0.001
*co*	1	0.18	0.674			
*t*	1	0.28	0.597			
*s × ca*	1	0.11	0.745			
*s × co*	1	0.28	0.594	1	14.06	<0.001
*ca × co*	1	3.21	0.073	1	11.73	0.001
*s × t*	1	2.79	0.095	1	4.54	0.033
*ca × t*	1	6.84	0.009	1	30.40	<0.001
*co × t*	1	0.19	0.661			
*s × ca × co*	1	0.13	0.716	1	7.00	0.008
*s × ca × t*	1	0.80	0.372			
*s × co × t*	1	0.04	0.850			
*ca × co × t*	1	2.64	0.104	1	12.06	0.001
AICc		421.9			403.4	

**Table 3:** Results of binary logistic regression of survival (Equation (3)) among cohorts in Armagh in 1988 (*s*: host species; *ca*: the presence of exclosure cages; *t* ordinal date). The reduced model is Equation (8).

	Full Model			Reduced Model		
Source	df	χ^2^	*p*	df	χ^2^	*p*
Regression	7	120.62	0.000	6	117.86	<0.001
*s*	1	27.89	0.000	1	38.36	<0.001
*ca*	1	3.82	0.051	1	34.83	<0.001
*t*	1	11.09	0.001	1	30.81	<0.001
*s × ca*	1	11.28	0.001	1	23.29	<0.001
*s × t*	1	6.76	0.009	1	10.16	0.001
*ca × t*	1	0.92	0.336			
*s × ca × t*	1	6.71	0.010	1	14.25	<0.001
AICc		320.7			318.50	

**Table 4:** ANOVA and Anderson-Darling residual normality tests of bird population density census data, $\sqrt{\mathrm{pairs}/{\mathrm{km}}^{2}}$. Significant F test triggered Bonferroni pairwise means comparisons in Figure 6a, separate for each bird species.

Species	F	*p*	AD	*p*	*n*
TEWA	236.1	<0.001	0.998	0.01	7
CMWA	227.4	<0.001	0.270	0.55	7
BLBW	6.4	0.08	0.126	0.97	7
BBWA	45.2	<0.001	0.129	0.97	7
Total	15.3	<0.001	0.151	0.93	7

**Table 5:** Two-way frequency table of bird activity observations. Row-wise tests compare specific activities between two sites.

Activity	BSL 1983	Armagh 1987	χ^2^	*p*
Singing and others	2264	1358	199.9	<0.001
Travelling	509	464	2.1	0.149
Searching	1737	2890	932.8	<0.001
Feeding	1438	252	754.1	<0.001
Overall			1270	<0.001

## Data Availability

The data used in this paper are available publicly at https://doi.org/10.23687/0cf57b1e-a136-48f3-a7b5-34749406dfae (accessed on 2021/08/09).
